# Development of a Computer-Tailored Intervention/Decision Aid To Increase Colorectal Cancer Screening in Health Systems

**DOI:** 10.7759/cureus.23372

**Published:** 2022-03-21

**Authors:** Hala Fatima, Maryiam Wajid, Connie Krier, Victoria Champion, Lisa Carter-Harris, Rivienne Shedd-Steele, Thomas F Imperiale, Peter Schwartz, Sylvia Strom, Mark Magnarella, Susan M Rawl

**Affiliations:** 1 Gastroenterology, Indiana University Health, Indianapolis, USA; 2 Internal Medicine, Indiana University School of Medicine, Indianapolis, USA; 3 Nursing, Indiana University School of Nursing, Indianapolis, USA; 4 Nursing, Memorial Sloan Kettering Cancer Center, New York, USA; 5 Health Services Research, Regenstrief Institute, Indianapolis, USA; 6 Community Advisory Board, Indianapolis, USA; 7 Senior Designer/CEO, Eo Studios, Athens, USA; 8 Cancer Prevention and Control, Indiana University Melvin and Bren Simon Comprehensive Cancer Center, Indianapolis, USA

**Keywords:** fecal immunochemical test, colonoscopy, colorectal cancer, screening, computer-tailored intervention

## Abstract

Background

Non-compliance with scheduled colonoscopy is common among patients, especially in underserved populations. High no-show and late cancelation rates result in wasted resources, increased costs, and missed opportunities for colorectal cancer (CRC) screening. Among the barriers to colonoscopy is a lack of knowledge about the benefits, fears, and limited time for patient counseling.

Methodology

We produced a digital video disc and a website program to enhance awareness about CRC screening and address patient barriers in a population with low screening adherence.

Results

Patients can be educated via an interactive computer-tailored intervention with both DVD and web versions. It details the benefits and need for CRC screening, different methods of screening, and addresses patient-related barriers.

Conclusions

Patient education is crucial to increase CRC screening among eligible individuals. Because online engagement is affected by attention, interest, and affect, content should be concise but comprehensive.

## Introduction

Colorectal cancer (CRC) is the second most common cause of cancer-related deaths in the United States [1]. The disease and its associated mortality can be prevented by early identification and removal of precancerous polyps [2]. The decline in CRC incidence and mortality is at least partially attributed to increased screening over the last several years [1,3,4]. The US Preventive Services Task Force recommends CRC screening for persons 50-75 years of age (grade A recommendation) [5]. In addition, its most recent recommendations include offering screening to adults aged 45-49 years (grade B recommendation). The American Cancer Society recommends screening at age 45 years in average-risk individuals [6], and the American College of Gastroenterology has recently adopted this recommendation as well [7]. Despite strong evidence favoring screening, more than a quarter of adults in the United States have not been screened as recommended [8]. In 2018, 68.8% of the adults aged 50-75 years were up to date with CRC screening across the United States. Although this was an increase from 67.4% in 2016, there remain 21.7 million people aged 50-75 in the United States who have never been screened [9].

CRC screening rates depend on multiple factors. Studies have highlighted lower rates of screening among Hispanics and African Americans compared to Whites [10,11]. African Americans have the highest incidence and mortality rates of CRC [1], highlighting the importance of recommending screening in this population, including those with lower socioeconomic status. Barriers to screening among African Americans include lack of knowledge about screening, lack of time, cost of a colonoscopy, fear of a cancer diagnosis, transportation issues, anxiety about colon preparation, lack of interest, and low perceived risk of cancer [12]. Similarly, in an exploratory study of Hispanic patients, barriers included cost due to lack of insurance, fear, and embarrassment, as well as lack of health education [13].

Research has shown that patient education, nursing interventions, mailed fecal occult blood test kits, and academic detailing lead to an increase in the uptake of screening [14-16]. Among these interventions, patient education is key to increasing uptake which can be delivered through healthcare providers, pamphlets, or multimedia. The advantages of providing patient education through multimedia are time efficiency, ease, affordability, and the minimal need for personnel [17].

Computer-tailored interventions (CTI) are effective approaches to increase CRC screening [18-24]. Although computer-tailored health communications are delivered using various media, computer technology is required for the tailoring process. Tailored interventions have influenced health behavior change with smoking cessation, dietary change, physical activity, mammography, and CRC screening [25-30].

We adapted an interactive CTI that was developed and shown to be effective for increasing CRC screening among African Americans [21]. In the current study, the intervention was revised to appeal to a racially/ethnically diverse audience to increase CRC screening among average-risk patients who did not attend their scheduled colonoscopy appointment. The intervention was produced in both English and Spanish and was made available in two delivery formats: (1) digital video disc (DVD) and (2) website program. The English version of the DVD format was evaluated among 123 participants in a comparative effectiveness trial funded by the Patient-Centered Outcomes Research Institute (IHS-1507-31333). Results showed that the DVD increased CRC screening by 10% compared to participants who did not receive it. This 10% difference was considered to be clinically important by expert clinicians on the research team. In this article, we describe the steps leading to the CTI so that its production may be replicated in other settings.

## Materials and methods

Intervention design and the development of the English version

The interactive CTI, “Time to ACT: Approaches to Colon Testing,” was a refinement of a tablet-based CTI previously developed to promote CRC screening among African Americans [21]. The intervention was refined over 11 months in 2016-2017 in collaboration with Eo Studios, an interactive multimedia design firm, our Community Advisory Board, and our research team which included gastroenterologists and experts in health communication. Message content was guided by the theoretical constructs of the Health Belief Model [31]. This model proposes that individuals will take action to prevent, screen for, or control a health condition if they: (1) consider themselves to be susceptible to or at risk for the condition; (2) believe the condition to have serious consequences; (3) believe that a particular course of action will reduce either their susceptibility to or the severity of the condition; and (4) believe that the anticipated barriers (costs) relative to taking the action are outweighed by the benefits [32]. Key content also centered around increasing confidence (i.e., self-efficacy). The goal of the CTI was to help participants make an informed decision about getting a CRC screening test and preparing for and completing that test. The program provided detailed information about screening colonoscopy as well as detailed information about performing a stool blood test (i.e., fecal immunochemical test or FIT) as an alternative screening test.

The program was introduced by Dr. Hala Fatima, Director of the Endoscopy Department at Eskenazi Health in Indianapolis, Indiana. Although tailored to this healthcare facility, the introduction is a module that can be replaced with a message from another healthcare system for translation into other practices. The underlying theme involved a doctor making a house call to provide information and encouragement to screen for preventing CRC. Filming took place in a home. A professional actor played the role of the doctor who narrated the entire program; therefore, minimal reading was required to engage with the program. The program required 20 minutes to view. Individual sections could be replayed using a chapter menu at the end of the module.

The first part of the program focused on how CRC develops, its risk factors, benefits of testing, and screening test options. A video described the anatomy and physiology of the colon, the development of polyps, and that polyps may turn into cancer. The risk of developing CRC was addressed by the doctor showing an animated graph where risk increased with age. Still images of men and women of different races and occupations were provided. The doctor continued to narrate as the user viewed graphs illustrating the good news that deaths from CRC had been cut in half over the past 40 years, in part, by more people getting tested for CRC. Next, a Public Service Announcement video played where Morgan Freeman urged people to get screened. Afterward, the doctor expanded upon the importance of screening because polyps and CRC can present with no symptoms. This was followed by a short testimonial from a woman who stressed the importance of regular screening and early testing if symptomatic because the cure rate is high.

The benefits of CRC testing were further emphasized by two powerful on-camera testimonials by CRC survivors who, like most people, did not think they would get CRC. One was a 50-year-old male firefighter who appeared healthy and physically fit. Although he had no symptoms, his colonoscopy revealed stage 3 cancer. The other testimonial was from a female educator who had symptoms but did not believe it could be CRC. After the testimonials, video clips described two of the most common CRC tests, colonoscopy and stool blood test (i.e., FIT). The description of colonoscopy began with a video clip of a doctor performing a colonoscopy and progressed to a video animation showing the pathway of the colonoscope and a polyp is removed. The video was also used to demonstrate that a patient was sedated during the procedure and that it was necessary to have someone drive the patient home afterward. For the stool blood test, the program demonstrated a mock sample collection which was filmed in the bathroom of the home.

The next section focused specifically on colonoscopy, and first included a tailored demonstration on completing the bowel preparation based upon whether split dosing of GoLYTELY/NuLYTELY or Miralax-Gatorade had been ordered. This was followed by an assessment of the participant’s emotional and logistical barriers to completing colonoscopy based upon identification of those barriers in our prior work [20-22,33-35]. Barriers assessed included: (1) embarrassment, (2) fear of colon injury, (3) pain, (4) cost, (5) bowel preparation, and (6) transportation. If a barrier was endorsed, a video of a CRC testing advocate or survivor delivering a tailored message to overcome the specific barrier played. The colonoscopy section concluded with the participant indicating if s/he intended to get a colonoscopy within the next six months or would like to hear more about the stool blood test.

**Figure 1:**
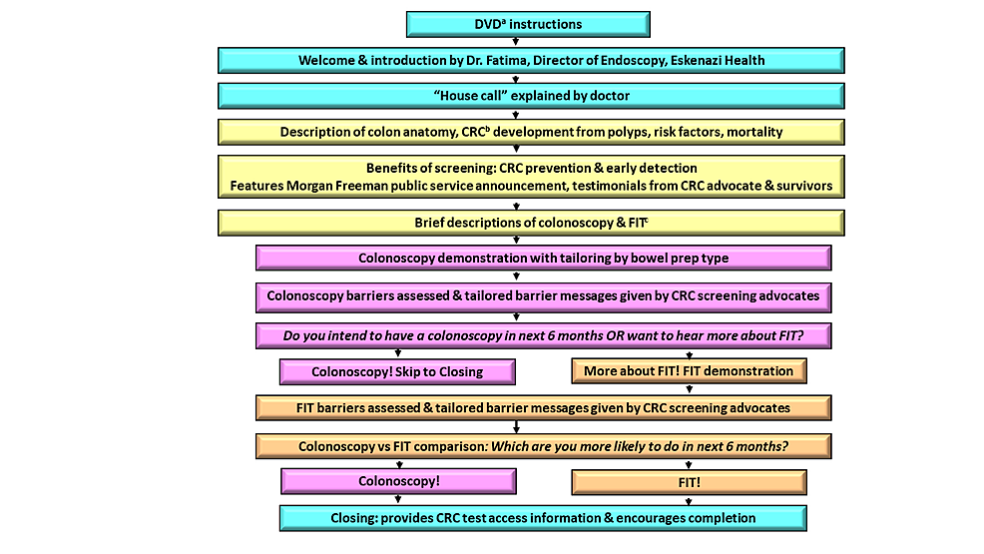
Content and flow of the computer-tailored intervention. DVD: digital video disc; CRC: colorectal cancer; FIT: fecal immunochemical test

The final section of the program included an in-depth description of FIT as a stool blood test and a reasonable alternative for average-risk screening. The video showed the contents of the FIT kit, how to prepare the toilet, how to use the sample collection brush, and how to return the FIT for lab testing. The program assessed common patient barriers to performing an FIT: (1) unpleasant, (2) embarrassing, and (3) cost. Similar to colonoscopy, if a barrier was endorsed, a tailored message to overcome the barrier was delivered. To help participants who might be struggling with which test to do, a summary of the advantages and disadvantages of each test was presented. The participant was then asked to select which test they were more likely to do in the next six months. Regardless of the test selected, the participant was reminded of the phone number to call to schedule the colonoscopy or get an FIT kit and encouraged to get tested soon. The content and flow of the CTI are illustrated in Figure 1. Roles in the development of the CTI are outlined in Appendix.

Once the DVD program was finalized, we built a web-enabled version for online accessibility. This component required approximately 10 weeks and increased the cost by 50%. Rather than being used in the comparative effectiveness trial, the web version was made available to the public at the end of the trial. This decision was made based upon the low number of study participants who were regular internet users with reliable service.

Our interactive learning-based design firm evaluated and selected PHP, MySQL, HTML5, JavaScript, and embedded VIMEO videos as the optimal web technologies for the program. The program was optimized for playing on the web such that each user received a low-, medium-, or high-resolution version depending upon their available bandwidth, allowing the videos to play without lagging. We selected VIMEO, a video specialist, to host and deliver the videos in the program so that they played smoothly across a range of devices and with minimal lagging due to VIMEO’s optimized content delivery network. We selected our university’s website to host the program. This associated the program with an easily recognizable and respected local research university without incurring a fee for service. We selected the domain name, mycolonhealth.iu.edu, for the web address. We designed a landing page for the website that included university branding, an introductory message, navigation instructions, and university-required elements such as contact information and a privacy notice.

Unlike a DVD, the web format allowed us to add an administrative database feature to capture user viewing and responses. This secure database recorded the user’s unique identification code, number of times the program was accessed, last log-in date, user responses, and program completion status. The data collected could also be exported to Excel and used for statistical analyses.

Design and development of the Spanish version

Due to the growing Hispanic population who are eligible for CRC screening at Eskenazi Health, a Spanish version of the interactive CTI was developed by our design firm after the trial ended. It was produced in both DVD and web formats to allow for broad dissemination and was consistent with the English version. Spoken and written content was translated into Spanish by a professional translation service and then reviewed by multiple native Spanish speakers for accuracy, quality, and “universal” comprehension (given the many Spanish dialects). Voiceover talent, based in the United States and Latin America, auditioned for four roles: male doctor, female doctor, female survivors, and male survivors. We selected two men and two women to deliver voiceovers for the entire program and matched their faster rate of speech to their English counterparts to loosely lip-synch the delivery (because Spanish translation is ~20-25% longer than the English counterpart). In addition, all titles, graphics, illustrations, and animations that included written content were translated and updated by the graphic designers, animators, and video editors. The text for the DVD packaging, which includes a full-color two-panel jacket and an on-disc label, was translated into Spanish. The homepage of the web version was updated to allow users to toggle (switch back/forth) between Spanish and English versions before starting the program in the preferred language. The time necessary to complete the Spanish version of the program was consistent with the English version at approximately 20 minutes.

## Results

The DVD was developed successfully. Before beginning recruitment for the comparative effectiveness trial, the CTI was tested in DVD format for usability and satisfaction with five individuals who represented our target population. They evaluated its ease of use, content level and appropriateness, aesthetic appeal, and relevance. Results were positive regarding ease of use and the importance of the information presented. All (100%) pre-testers indicated that they would recommend this program to others.

**Figure 2:**
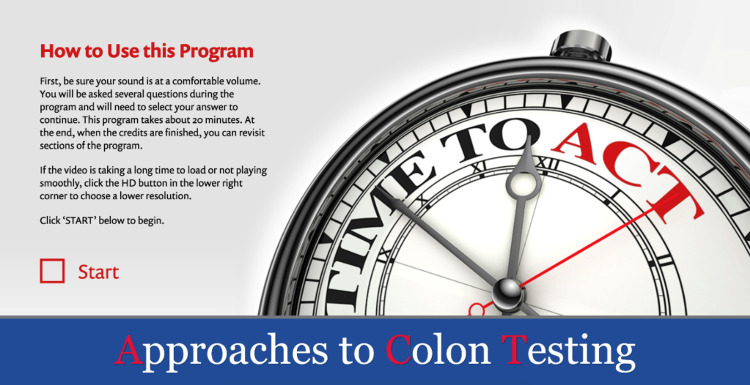
Instructions on the use of the program.

**Figure 3:**
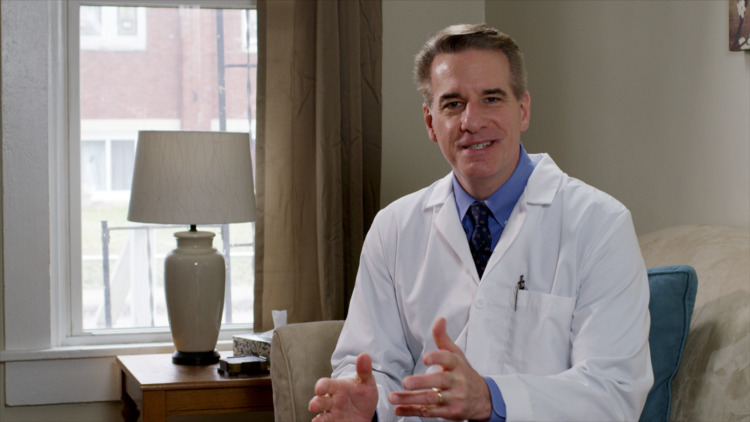
House-call theme.

**Figure 4:**
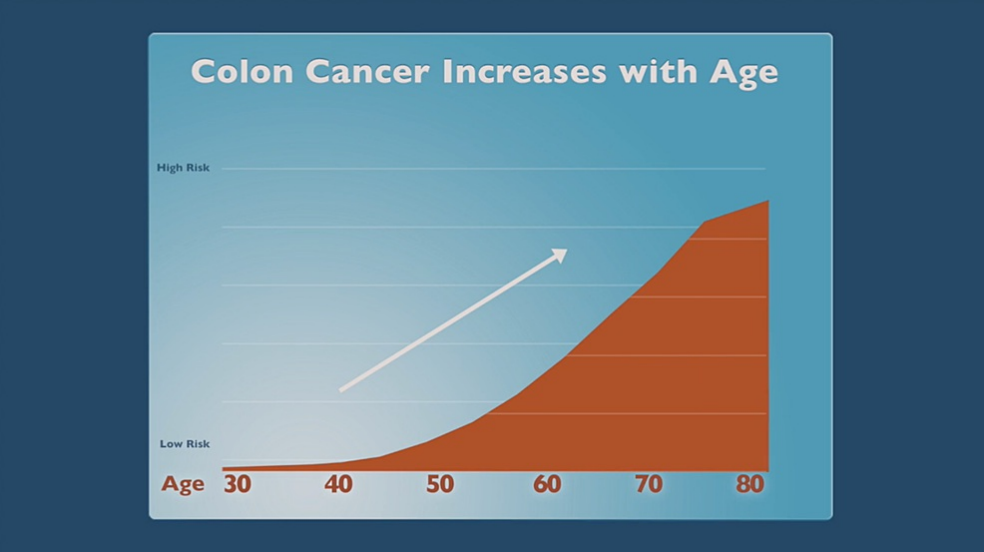
Knowledge: risk of colorectal cancer.

**Figure 5:**
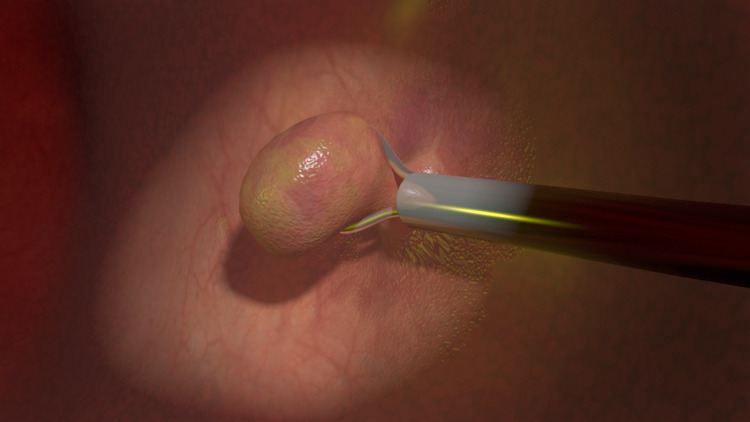
Polyps removed during a colonoscopy.

**Figure 6:**
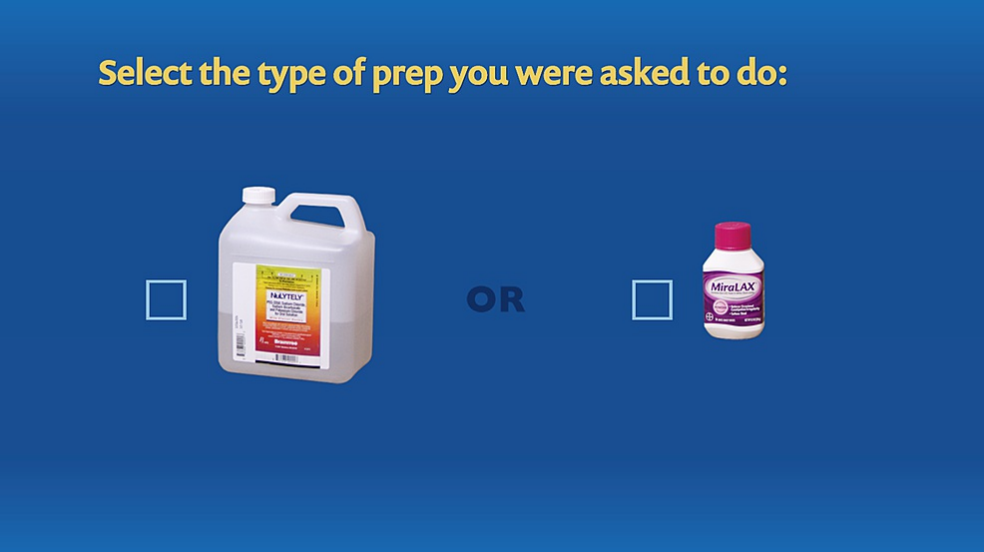
Tailoring variable: type of bowel preparation ordered by the doctor.

**Figure 7:**
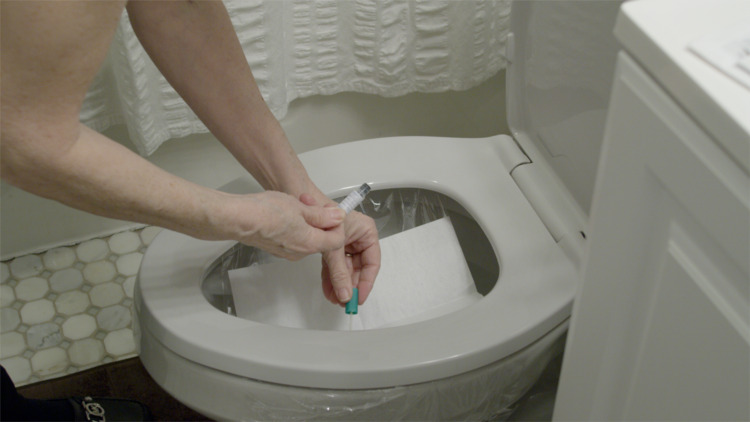
Colorectal cancer testing by a stool blood test: fecal immunochemical test.

Sample images from the DVD regarding patient education regarding the risk for CRC and modes of screening are shown in Figures 2-7.

**Figure 8:**
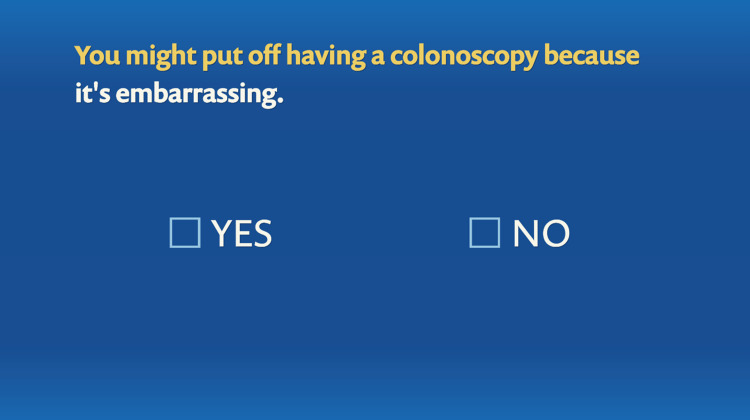
Tailoring variable: embarrassment as a barrier to colonoscopy.

**Figure 9:**
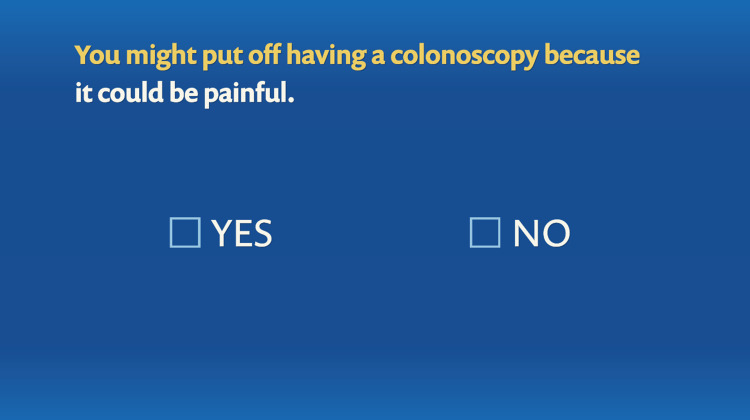
Tailoring variable: pain as a barrier to colonoscopy.

**Figure 10:**
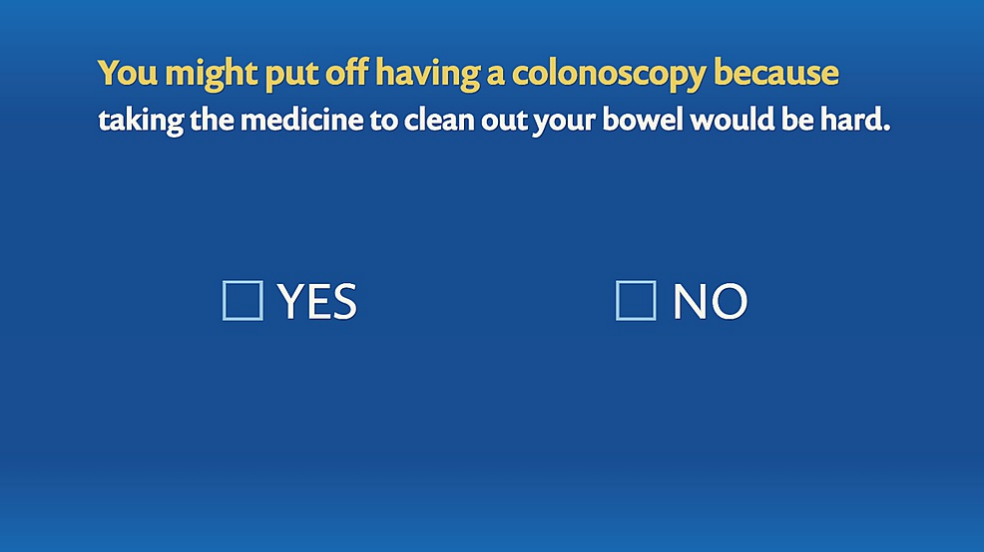
Tailoring variable: bowel preparation as a barrier to colonoscopy.

**Figure 11:**
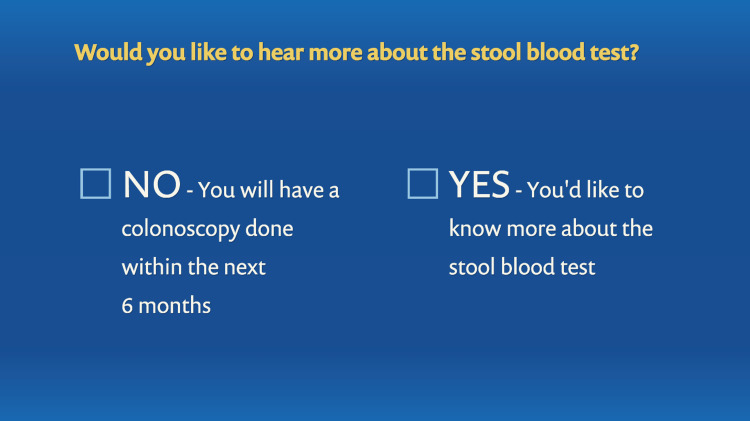
Tailoring variable: colonoscopy versus stool blood test.

The program was interactive and allowed the users to toggle between menus to learn about colonoscopy and stool-based testing for CRC screening. It also allowed them to click through various barriers to screening and learn about solutions. In addition, there were patient testimonials that were helpful in dispelling these barriers. The interactive nature of the program is illustrated in Figures 8-11.

## Discussion

Most endoscopy units are open access which means patients usually get directly referred for colonoscopy for CRC screening by their primary care physicians or family physicians. Patients, however, remain uninformed about many aspects, including the importance of screening, alternatives, risk information, and eliciting barriers. Patients also may not be given information about stool-based screening tests. In addition, most referring physicians do not respond adequately to patients’ concerns about screening, which leads to increased anxiety and stress related to the procedure and hence low uptake of screening. This is especially true for patients from lower socioeconomic status and particularly if there is a language barrier [36].

There are several barriers to CRC screening [37], including patient and physician factors. Patient factors include embarrassment, discomfort, fear of test, fear of finding cancer, privacy concerns, lack of knowledge, distrust of healthcare system, transportation issues, childcare issues, lack of time, and lack of insurance, among others. Physician factors include lack of physician recommendation, lack of explanation, inadequate patient-provider communication, and lack of time. These barriers are even more pronounced in African American and minority populations and add to the screening healthcare disparities [38]. Some of these barriers can be addressed through better patient education.

The optimal solution would be to have screening clinics where a physician/nurse practitioner discusses the importance of screening, explains the colonoscopy procedure and preparation process, answers any questions, dispels myths, describes how to complete the FIT test for patients who refuse colonoscopy, and discusses the pros and cons of each screening method. In addition, barriers around transportation and insurance can be handled by a caseworker. However, this is not a feasible option given the number of screening-eligible patients and the cost of providing this service. DVD education is a viable and cost-effective option. It has several advantages including the easy understanding of educational materials and interactivity to help seek solutions to problems. In addition, it explains both colonoscopy and FIT as viable options for screening. According to 2011 data from the Healthcare Effectiveness Data and Information Set, 62-64% of age-eligible patients who received an educational DVD had appropriate CRC screening compared to 44-48% who got routine instructions [39]. It has been shown that traditional instructions for bowel preparation may not be enough [40,41], and enhanced patient education using multimedia and visual aids can lead to better bowel preparation and increase the adenoma detection rate [42,43].

Screening colonoscopy can be stressful, especially if the details of the procedure are unknown to the patient. Educating patients about what to expect before and during the colonoscopy can reduce the stress of the unknown. A recent randomized controlled trial assessing the use of DVDs for coronary angiography showed that it is more effective than conventional pamphlets in controlling anxiety and stress [44]. There are patient-associated barriers particular to accessing the educational material on a DVD, including lack of access to a DVD player, unfamiliarity with usage, and inability to use the interactive features. In addition, there are logistic drawbacks to using a DVD as an educational tool, such as (1) content could become quickly outdated with changes in bowel preparation procedures and screening recommendations; (2) extra costs for a DVD label and case; and (3) increased pricing for reorders and shipping which may occur over time.

Given the popularity of the internet as a mode of online education, having the contents of a DVD as a web version is ideal. A link to the web version could be added to the patient’s electronic medical record portal for easy access and viewing. In 2021, 83% of Americans own a smartphone according to the Pew Research Center. Therefore, developing a smartphone application would increase the reach of the intervention. Web formats allow program content to be updated to remain current. There will be costs, however, to make these updates. Other common costs for a web-based program include costs to use a video streaming service, website hosting fees, and website routine maintenance charges. It is important to design website architecture to provide information in packages that are both small and interesting to keep the participant engaged and to avoid overwhelming them with bulky content. This is especially important among participants with lower educational levels [45].

## Conclusions

Patient education is very important to increase CRC screening among eligible individuals. We developed an interactive CTI to deliver this information. It details the benefits and need for CRC screening, different methods of screening, and addresses patient-related barriers. Although developing a CTI in the DVD or web format may be time-consuming and costly, it is substantially more cost-effective compared to the treatment of advanced CRC if screening is not performed. A web version may be a more attractive option compared to the DVD version given better availability, accessibility, and ease of updating content. Online engagement Is affected by attention, interest, and affect; therefore, content should be concise but comprehensive.

## Appendices

**Table 1:** Roles in the development of the computer-tailored intervention. CRC: colorectal cancer; FIT: fecal immunochemical test; DVD: digital video disc

DVD component	Research team	Community Advisory Board (CAB)	Eo Studios
“House call” theme developed	ü	ü	
Script written	ü		
Professional actor selected for doctor role	ü	ü	
CRC survivor and advocates selected for testimonials and barrier messages	ü	ü	
Filming on location: home, and at Eskenazi	ü	ü	ü
Selected and added video animation: IU-produced animation of polyp and cancer development; colonoscope in the bowel. Procured animation from other sources: ACS/Amgen, U of Utah YouTube video	ü	ü	ü
Added Morgan Freeman Public Service Announcement (used in an earlier study)	ü	ü	ü
Created animated charts: CRC risk increasing with age and CRC mortality decreasing in previous years. Pie chart with a 90% cure rate appeared in an earlier study	ü		ü
Selected still images	ü	ü	ü
Selected text to appear on the screen: barriers, table comparing colonoscopy to FIT, etc.	ü		ü
Built and implemented interactive components			ü
Incorporated tailoring algorithms			ü
Incorporated navigation elements into viewing formats: remote control for DVD; mouse for web			ü
Selected background music	ü	ü	ü
Designed DVD label and sleeve	ü	ü	ü
Replicated into DVD format			ü
Added web version to IUPUI-hosted website			ü
